# The Relationship between Impulsivity and Internet Addiction in Chinese College Students: A Moderated Mediation Analysis of Meaning in Life and Self-Esteem

**DOI:** 10.1371/journal.pone.0131597

**Published:** 2015-07-14

**Authors:** Ying Zhang, Songli Mei, Li Li, Jingxin Chai, Jiaomeng Li, Hongyang Du

**Affiliations:** 1 Department of Education, The First Affiliated Hospital, Liaoning Medical University, Jinzhou, China; 2 Department of Children and Adolescent Health Care, School of Public Health, Jilin University, Changchun, China; 3 Department of dermatology, The First Affiliated Hospital, Liaoning Medical University, Jinzhou, China; University of Ariel, ISRAEL

## Abstract

Internet addiction (IA) has increasingly been recognized as a serious psychological malady among college students. Impulsivity has been shown to be associated to addictive behaviors, also to IA, and that the purpose of the study is to investigate whether or not there are variables modulating the relation between impulsivity and IA. “Meaning in life” is regarded as a desirable attribute, with positive mental health outcomes. “Self-esteem” is often regarded as an important component of psychological health which has relation to IA. Therefore, we examined meaning in life and self-esteem’s possible effects in this relationship. A total of 1068 Chinese college students ranging in age from 18 to 25 years were recruited for this cross-sectional survey study. Correlations and multivariate regressions were used to calculate the possible mediation and moderation relationship among the variables of meaning in life, self-esteem, impulsivity, and IA. In the analyses that we conducted, IA was shown to be prevalent among Chinese university students. The relationship between impulsivity and IA was partially mediated by meaning in life, and the relationship between meaning in life and IA was moderated by self-esteem. Our findings demonstrate that meaning in life and self-esteem can be useful buffers to IA for highly impulsive individuals. Further randomized trials to confirm these results are needed.

## Introduction

Recently there has been explosive growth in the popularity of the internet at different stages of life. College students, who are at a critical developmental stage, warrant examination in this respect, because each has a laptop which initially facilitates academic activities, but later is available for recreational activities. Addiction to the internet is becoming a serious mental health issue and strongly impacts academic performance in college samples [1, 2]. Internet addiction (IA), appears to be a relatively common behavioral addiction, has certain symptoms and will undergo the same consequences brought about by addiction to alcohol and drugs as well as other obsessive behaviors [3]. Understanding how college students resist or fail to resist this temptation might shed light on internet addiction. Much research has been conducted in the account for what result in or follows from internet addiction, such as decision making, shyness, social anxiety, depression, conflictual family relations [4–7], and prefrontal control, dysfunctional inhibitory control, impulsivity [8, 9].

Impulsive individuals have problems in managing their behavior. Some core indicators of behavioral addiction are identical to those of chemical or substance addiction [10]. Behavioral addiction, such as IA criteria are presented firstly is recurrent failure to resist impulses to engage in a specified behavior [11], and a feeling of lack of control while engaging in the behavior. A large body of the literature in this area concerns impulsiveness impacting the addictive tendencies [12–14]. Both behavioral and substance addictions are marked by an inability to stop. One of the most valid methods of intervening those addictions is by identifying and reducing the negative catalysts and strengthening the positive aspects. This pertains much to high impulsivity in addicts, also to internet addiction [15]. As impulsivity might not be greatly decreased by psychotherapeutic intervention [16, 17], other psychological variables may instead be more effective in reducing it. Perhaps impulsive individuals might also possess protective qualities that control them from becoming preoccupied with the internet. We sought to investigate whether or not there are variables modulating the relation between impulsivity and IA, and furthermore, to address the psychological strengths that predict one’s ability to resist the constant allure of the internet. To this end, we examined two complementary mental strengths in resisting IA: meaning in life, and self-esteem.

What kinds of psychological features do people have when they are overly involved in use of the internet? Meaning in life is found to have a stronger positive association with psychological well-being [18, 19]. Meaning in life as an association between psychosocial well-being and internet addiction has received much more attention and consistent empirical support [20, 21]. Meaning in life is typically referred to as a sense of purpose in one’s life and an accompanying sense of fulfillment [22, 23]. It is a desirable attribute in the realm of attitude evaluation. When they confront temptation, they have control over the outcomes, affecting them in important ways. Finding meaning in life means that people feel positive and efficacious, as a psychological strength [22, 24].

An decreased level of meaning in life has been correlated with several substantial addictive behaviors among individuals [25]. Meaning in life plays important role in treating people with alcoholism use [26]. Meaninglessness in life sometimes brings about a mediating effect on adolescents’ life events and substance used among it [27]. Having a weaker sense of meaning and purpose in life results in an increased proneness to boredom and empty life [28, 29]. Meaningfulness is a cognitive model of empowerment as the basis for worker empowerment [30]. Boredom was common trigger of intensive internet use [31]. This in turn, might increase the probability of internet addiction, especially for students at universities where surfing the internet is relatively easy and socially accepted. Consequently, it is important to determine whether meaning in life provides a buffer against addictive behavior in college students.

The essence of human motivation is the “will to meaning” [32], and a sense of meaning as a cognitive factor has been identified as a potential protective component for individuals [33]. Thus, while purpose or meaning in life confers benefits for enjoyment of work and positive life attitudes, what happens when people are confronted with the constant allure of the internet? Perhaps meaning in life only provides resiliency when individuals also possess characteristics that allow them to resist hedonism. Self-esteem may be such a characteristic. It may complement meaning in life to confer resistance to IA.

Self-esteem is a psychological strength reflecting one’s overall evaluation of oneself. High self-esteem is often regarded as an important component of psychological health [34, 35]. There is some debate over whether self-esteem is more trait-like or state-like [36]. For the purposes of our study, we considered self-esteem as a stable trait factor because we used an adult sample [37]. Given self-esteem’s broad implications, it seems a distinct possibility that high self-esteem impacts the pursuit of long-term goals with well-anchored, positive attitudes about the self and happiness [38]. A close relationship has been documented between low self-esteem and problems such as alcoholism, drug abuse, eating disorders, school dropouts, poor academic performance, pregnancy in adolescence [39–42].

There are several studies on the relevance of self-esteem to internet addiction [43, 44]. One study found that emotional support from both parents would increase their child’s self-esteem, which in turn would reduce the child’s risk of being addicted to the internet [45]. Additionally, research has shown that self-esteem is one of the main antecedents of IA [46]. Individuals with lower self-esteem are more likely to be addicted to the internet [47, 48].

Further, there are several reasons why high self-esteem may be a source of resiliency. First, compatible with theories of self-esteem’s evaluation function and emotional experience [49], “high self-esteem” individuals are more likely to evaluate themselves as having positive self-esteem and self-competence. Individuals with positive self-value could generate and strengthen their degree of meaning in life. Though these people are lured by the internet as much as others, they may be more likely to maintain or promote their efforts toward attaining meaningful goals instead of aborting them. Second, some studies support the buffer hypothesis, that high self-esteem enhances initiative and pleasant feelings [50, 51]. “High self-esteem” confers personal ability and value, so it may serve as a positive factor against addiction which allows individuals to create and realize life goals and purpose in life. One of self-esteem psychological structure just is meaning in life [52]. In combination, the variables of meaning in life and self-esteem might confer optimal protection from internet addiction. The meaningful pursuits of “high self-esteem” individuals allow them to bridge the gap between their real and ideal selves.

## Purpose of the Present Study

In this study, we examined the influences of meaning in life and self-esteem on internet addiction. We argue that meaning in life and self-esteem, each account for impulsivity individual addictive behavior to some degree. To date, the hypothesis that self-esteem moderates the relationship between meaning in life and internet addiction has not been tested. This is the first study to test a synergistic effect between a life attitude (meaning in life) and a self attitude (self-esteem) for a more sophisticated model of resiliency. As mentioned previously, we hypothesized that (H_1_) meaning in life mediated the relationship between impulsivity and internet addiction. Based on theoretical models of why people indulge in the internet [45, 50], we hypothesized that (H_2_) the relationship between meaning in life and internet addiction is moderated by self-esteem. This allowed a test of construct specificity to IA under more constricted methodological factors.

## Methods

### Ethics Statement

This study was a cross-sectional survey, and data collection occurred within the context of a larger lecture on mental health. The research was approved by the Institutional Review Board (IRB), School of Public Health, Jilin University, China. All participants gave written informed consent, and were ensured complete anonymity. Participation was voluntary, and students were given information about campus mental health resources upon completing the study.

### Participants

We tested our hypotheses on a large random sample of college students (*N* = 1537). All participants were students of medical disciplines at three colleges in North China. They completed self-report measures in the classroom after a formal lecture. A total of 1068 eligible participants (61.1% female; mean age = 22.76, *SD* = 2.54, range 18–25) responded to the present study. The participation response rate was 69.5%. This rate is similar to those of previous studies [53, 54]. There were no significant interactions with gender and socioeconomic status (*p* > .05), so these factors were not included in further analyses.

### Questionnaires

Participants completed paper-and-pencil self-report questionnaire packets and measures of demographic information, impulsivity (BIS-11), meaning in life (PIL), self-esteem (RSE), and internet addiction (YDQ).

IA was computed from responses on a widely-used eight-item Internet Addiction Diagnostic Questionnaire (YDQ). The YDQ pertains to all types of online activity and has no time limit. Respondents who answered yes to five or more of the eight criteria were classified as addicted internet users [55]. We considered lower general scores as an indicator of higher IA. The YDQ has been deemed valid and reliable in previous research reports [56].

Impulsivity was measured using the Barratt Impulsiveness Scale 11 (BIS-11). The BIS-11 is a questionnaire on which participants rate their frequency of several common impulsive or nonimpulsive behaviors/traits on a scale from 1 (rarely/never) to 4 (almost always/always). The questionnaire consists of thirty items, the minimum score is 30, and the maximum is 120; with higher scores indicating greater impulsivity. Its reliability and validity have been shown to be acceptable [57].

Meaning in life was measured with the widely used Purpose in Life Test (PIL) [58], an attitude scale which includes twenty ratings made on a 7-point scale, where a “1” indicates low purpose and a “7” indicates high purpose, the minimum score is 20, and the maximum is 140. The PIL provides participants with unique anchors for each item. Some of these anchors are bipolar, some are unipolar, and some utilize a continuum. For example, one item provides a continuum on which one end is “If I could choose, I would prefer never to have been born.” On the other end of the continuum is “If I could choose I would live nine more lives just like this one.” The scale has generally demonstrated good internal consistency [18].

The Rosenberg Self-Esteem scale (RSE) was used to measure overall feelings of self value and self acceptance [59]. Participants rated their level of agreement with ten statements on a 5-point Likert-type scale ranging from 1 (strongly disagree) to 5 (strongly agree). Sample items include, “I feel that I have a number of good qualities,” and “All in all, I am inclined to feel that I’m a failure.” The minimum score is 10, and the maximum is 40. Scores were computed by averaging the items, with higher scores indicating higher self-esteem. This instrument is a well-validated and reliable measure of global self-esteem [60].

## Statistical Analysis

### Preliminary analyses

Analysis of the data from this study was performed using SPSS 21.0 statistical software (SPSS, IBM, Lmd, Beijing, China). Missing values in the data were computed along with the sample means. Internet addicts in the general population have a relatively low base-rate occurrence and we found evidence of negative skewness in our sample (skewed = -1.461, *SE* = .075). Due to a large sample size (*N* = 1068), all variables were sufficiently normally distributed [61]. Bivariate scatter plots showed linear relationships between all variables, and we used stepwise linear regression analyses. Significance values were set at *p* < .05. Correlations were calculated between the variables of self-esteem, meaning in life, impulsivity, and internet addiction. Values for Cronbach’s alpha are reported in Table 1.

**Table 1:** Means, standard deviations, intercorrelations, and internal consistency between study variables.

	1	2	3	4
**1 Internet addiction**	—			
**2 Impulsivity**	-.781**	—		
**3 Meaning in life**	.846**	-.751**	—	
**4 Self esteem**	.723**	-.546**	.647**	—
***M***	14.58	71.19	94.84	28.90
***SD***	1.742	9.802	13.617	4.131
***α* coefficient**	0.730	0.787	0.795	0.789

Note: N = 1068;

** *p* < 0.01.

### Mediation and moderation analyses

**Fig 1:**
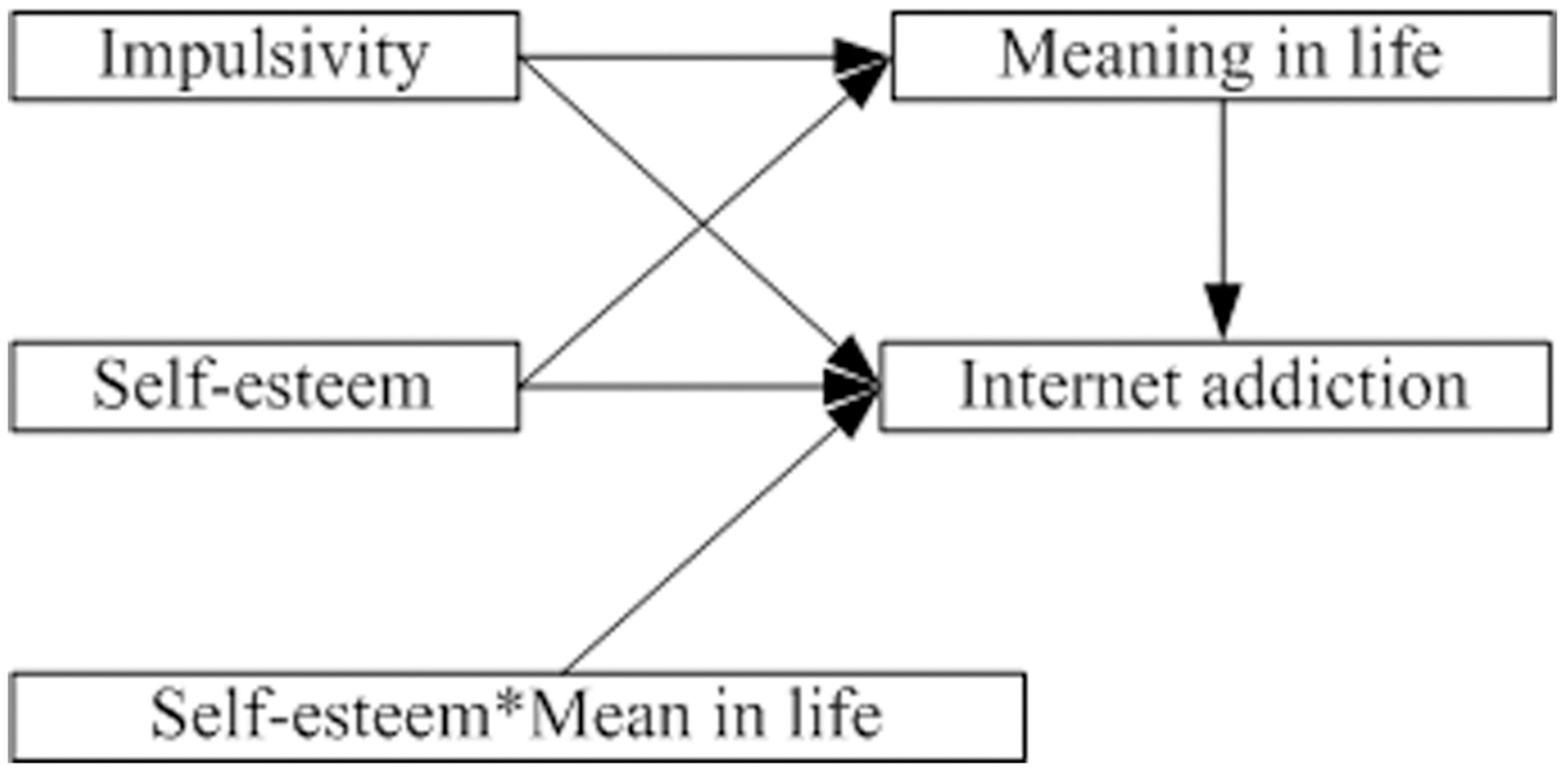
The conceptual framework.

As outlined in the Introduction, we conducted a specific mediation and a specific moderation hypothesis. As shown in Fig 1, we tested the extent to which the relationship between impulsivity and internet addiction is mediated by meaning in life, as well as the extent to which the relationship between meaning in life and internet addiction is moderated by self-esteem. To this end, we tested mediation by finding four regression coefficients: the total effect of impulsivity on internet addiction (c), the direct effect of impulsivity on internet addiction (c'), the effect of impulsivity on meaning in life (a), and the effect of meaning in life on internet addiction (b). When a, b, and c effects are significant, but the c' effect is not significant, a total mediation effect is shown. When a, b, c and c' effects are all significant, a partial mediation effect exists.

Next, we tested moderation effects by implementing the hierarchical regression technique. In the first regression, internet addiction was regressed on impulsivity and self-esteem. The coefficient for impulsivity was significant. In the second regression, meaning in life was regressed on impulsivity and self-esteem. The coefficient for impulsivity was significant here too. In the third regression, internet addiction was regressed on all predictor variables (impulsivity, self-esteem, and meaning in life), and the coefficient for meaning in life was significant. In the final step, internet addiction was regressed on impulsivity, self-esteem, and meaning in life, and the interaction between meaning in life and self-esteem. If the coefficient for the interaction between meaning in life and self-esteem is significant, moderated mediation has occurred [62–66].

Last, an interaction term was created by centering meaning in life and self-esteem around their grand means, and then multiplying them to avoid issues of collinearity with the interaction term. The main effects of meaning in life and self-esteem that is reported are the centered variables. The means and standard deviations reported are from the uncentered variables [67, 68]. Next, multiple regressions were conducted. Meaning in life (main effect) and self-esteem (main effect), were entered in Block 1 of the regression analysis while the interaction term (meaning in life × self-esteem) was entered in Block 2 when predicting internet addiction. If the interaction term significantly predicts internet addiction, a moderation effect has been found. To interpret the moderation effect, the data were entered into the regression equation based on high (1 SD above) and low (1 SD below) values of the moderator and mediator variables. In addition, a post-hoc probing of the interaction was conducted using two new conditional interaction terms (high and low) [69]. This was done to determine if the slopes of the regression equations for high and low values of the interaction differed from zero.

## Results

### Descriptive statistics

Table 1 displays the means, standard deviations, and correlations between study variables. All variables were significantly and positively correlated in the expected direction. The results indicate excellent reliability for the YDQ, BIS-11, PIL, and RSE questionnaires. Approximately 7.6% of the sample manifested clinically relevant levels of internet addiction. These rates are comparable to, and even above what might be expected. A recent cohort study of college students indicated that 74.5% were moderate users, 24.8% were possible addicts, and 0.7% were addicts [70]. Variance inflation factors of all predictor variables included in the regression analysis varied between 1.0 and 2.2, indicating that multi-collinearity between predictor variables did not bias our results.

### Mediation of meaning in life between impulsivity and internet addiction

**Fig 2:**
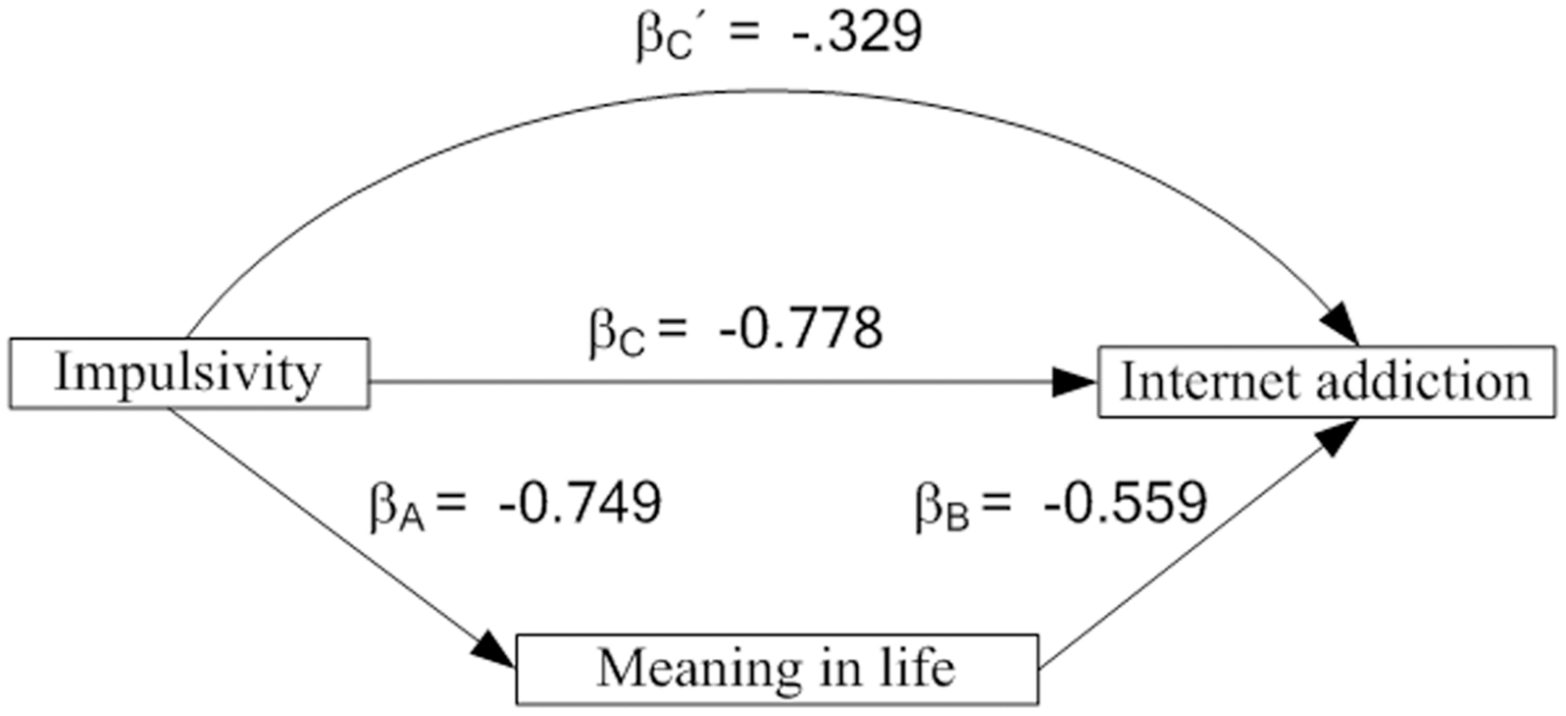
Standardized regression coefficients for paths within the mediation model . Explained variance: β, standardized regression coefficient.

Table 2 shows the results of the three separate regression analyses testing the mediation hypothesis. In step 1, a significant effect of impulsivity on internet addiction resulted (*b* = -.139, *p* < .001). In step 2, the effect of impulsivity on meaning in life was also significant (*b* = -1.403, *p* < .001). In step 3, after including the mediator variable meaning in life as a predictor in the regression model, the effect of both meaning in life and impulsivity on internet addiction was highly significant. Meaning in life was thus a significant part mediator (*△R* ^*2*^ = .606, *p* < .001)of the association between impulsivity and the outcome variables. This significant mediation effect is depicted in Fig 2.

**Table 2:** Summary of hierarchical regression analysis for meaning in life in mediating the relationship between impulsivity and internet addiction.

	*B*	*SE*	*t*
***PATH c (Step 1)*Internet addiction**	-0.139	-.003	-40.349***
***PATH a (Step 2)*meaning in life**	-1.403	-.028	-37.081***
***PATH c’+ b (Step 3)*Internet addiction**	Y = .076w -.059x	.003.004	26.527*** -14.577***

Note:

*** *p* < 0.001

Explained variance: step 1, *R* ^*2*^ = .606; step 2, *R* ^*2*^ = .561; step 3, *R* ^*2*^ = .763; *B*, unstandardized regression coefficient; *SE*, standard error of *B*.

### Moderation of self-esteem and meaning in life on internet addiction

Table 3 shows self-esteem as a moderator of the path from meaning in life to internet addiction, which was tested by the regression analyses just discussed. We controlled for age and gender here, and paths are labeled with coefficients from the regression equations used to estimate the predictor variables. First, internet addiction was predicted by the moderated variable and independent variable (β = -.55, *p* < .001). Subsequently, meaning in life was predicted by impulsivity and self-esteem (β = -.56, *p* < .001). Then, internet addiction was predicted by impulsivity, self-esteem and meaning in life (β = -.46, *p* < .001). Last, internet addiction was predicted by the moderated variable, meaning in life, self-esteem and meaning in life*self-esteem (β = -.25, *p* < .001). The R-square value changed due to the introduction of the interaction term in the analysis (*△R* ^*2*^ ≤ .05). The significant interaction effect supported our hypothesis of moderated mediation.

**Table 3:** The buffering effect of self-esteem on the relationship between meaning in life and internet addiction.

Step	1		2		3		4	
Predictor	Internet addiction		meaning in life		Internet addiction		Internet addiction	
	*b*	*t*	*b*	*t*	*b*	*t*	*b*	*t*
**Impulsivity**	-.10	-29.39***	-.78	-26.06***	-0.05	-14.33***	-.04	-13.87***
**Self-esteem**	.178	22.48***	1.12	15.69***	0.11	15.03***	.09	14.26***
**meaning in life**					0.81	20.42***	.78	23.16***
**meaning in life × Self-esteem**							-.09	-19.83***
***△R*** ^***2***^							0.05*	

Note:

*** *p* < 0.001,

* *p* ≤ 0.05.

### Meaning in life and self-esteem as synergistic factors

**Fig 3:**
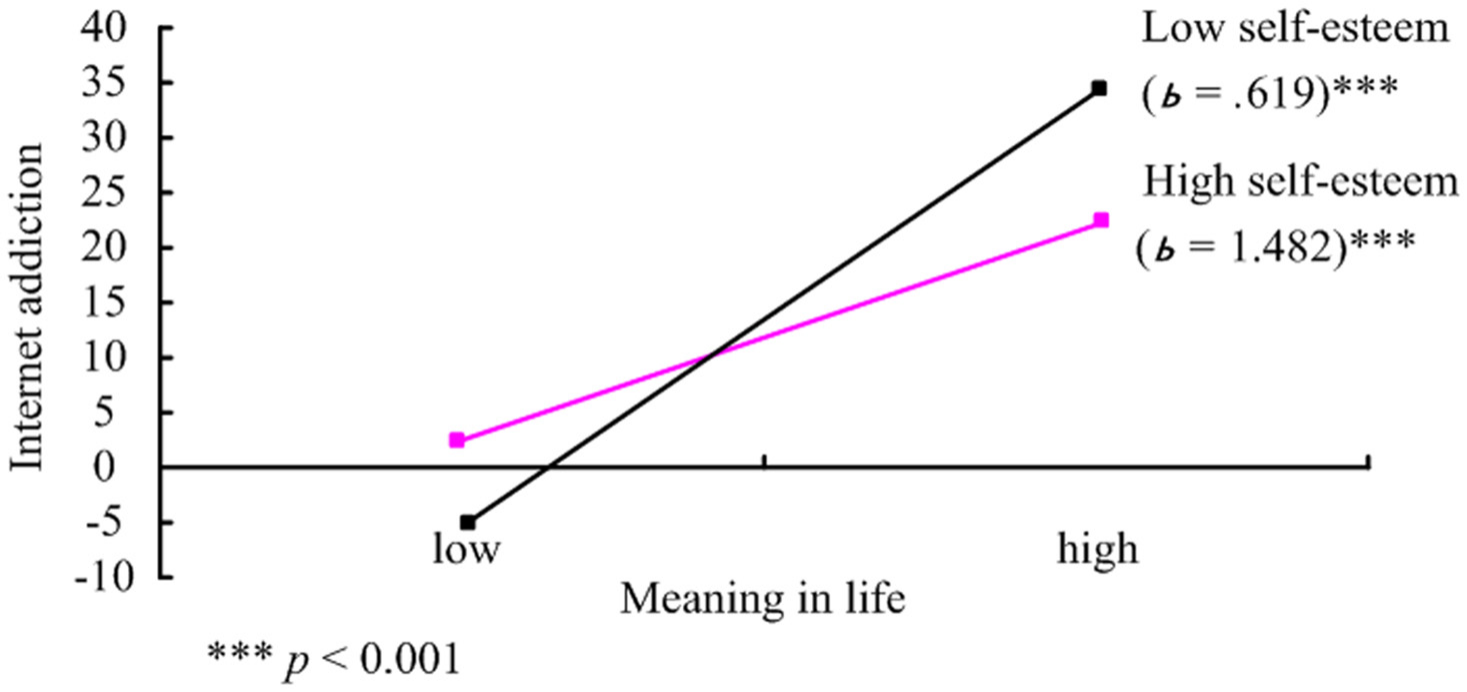
Regression lines for relations between meaning in life and Internet addict as moderated by self-esteem. (a 2-way interaction). *b* = unstandardized regression coefficient (i.e., simple slope); *SD* = standard deviation.

Given that there was an interaction between meaning in life and self-esteem on one’s likelihood of internet addiction, post-hoc analyses were conducted and plotted [68, 71]. Fig 3 shows the results of these analyses. As meaning in life increased, internet addiction decreased. It is clear from the beta values for the prime condition that meaning in life positively predicted internet addiction scores for participants in both the low self-esteem and high self-esteem conditions. However, the relationship between meaning in life and internet addiction was stronger for those with low self-esteem than for those with high self-esteem. When meaning in life is low, low self-esteem has a substantial effect on internet addiction. Results from the post-hoc probing indicate that the slopes for high and low values of the moderator were significantly different from zero, further supporting moderation.

## Discussion

The results of the present study reveal the relationship between several variables. One such relationship is the significant direct effect of impulsivity on internet addiction. Our finding is in line with numerous studies that demonstrate that individuals with higher trait impulsivity are more likely to engage in impulsive internet use [15, 72].

The present research also supported that the effect of impulsivity on internet addiction is partially mediated by meaning in life. Partial mediation such as that found here is common and accepted in the behavioral sciences, as complete mediation is quite rare in this domain. There is theoretical support for depression, anxiety, lower for self-directedness and cooperativeness variables pathways to impulsive addictive behaviors. The nature of the relationship between impulsivity, meaning in life, and internet addiction is complicated. Meaning is recognized as a central human motivation [73], and living a meaningful life is associated with positive functioning [74]. For instance, purpose in life predicts better emotional recovery from negative stimuli [75]. However, meaning in life may be more distal as a protective factor.

College students are in a key life stage centered around the search for meaning and establishing self-identity [76]. Finding meaning in life is a critical dilemma for these students undergoing great mental and behavioral development. Students who were retaking the college entrance examination experienced a positive life change and found greater meaning in university life [77]. However, those with less impulse control and meaning in life may be more susceptible to problematic behavior.

Among the vast array of psychological variables, we chose self-esteem and meaning in life as our focus for important reasons. First, meaning in life is an attitude pertaining much to the external environment, while self-esteem is an internally-oriented evaluation of oneself. Self-esteem is a critical component of any self-improvement or rehabilitation program. Furthermore, high self-esteem individuals have an internal characteristic that to an extent, helps them resist addictive behaviors. An individual with both of these complementary strengths may be the most safeguarded from internet addiction. Second, self-esteem not only affects our values, but our feelings and actions in a variety of circumstances [78]. While meaning must be based on one’s daily experiences, we infer that high self-esteem can moderate a sense of purpose and meaning in life.

However, it is also necessary to understand other factors from which improper behaviors arise during this dynamic period of development. The self-esteem pathway has received much more attention and consistent empirical support. Prior work suggests that low self-esteem individuals are more likely to be identified as internet addicts [79]. Individuals at greatest risk for internet addiction possess a combination of boredom with leisure activities and other psychological traits such as self-exclusion, and identity problems. Self-esteem fits in well to the cumulative continuity principle of personality development because the consistency of self-esteem increases with age [37].

With the growth of positive psychology, the variables of meaning in life and self-esteem have received great attention [51, 80]. They may confer synergistic benefits in combating internet addiction. Studying co-occurring protective factors offer a broader understanding of how one factor changes the effect of another on a given behavior. Risk and protective factors are related, but are not interchangeable [81]. We suggest that people can circumvent internet addiction with resiliency factors. Since meaning in life and self-esteem could both be enhanced in positive psychotherapy [82, 83], we present this study as a model to describe possible moderators and mediators that may work in combination to restrain overindulgence in the internet. Our findings also suggest that individuals with high baseline levels of both strengths showed significantly decreased internet addiction relative to their counterparts without these strengths combined.

Taken together, the results reported here imply that meaning in life and self-esteem offer substantial protection against internet addiction. Our findings extend previous research by exploring additional psychological buffers against internet addiction, such as perceived social competence [84]. To our knowledge, this study is the first to examine meaning in life as it applies to internet addiction. Despite evidence for an inverse association between self-esteem and internet addiction, meaning in life predicted internet addiction only in combination with high levels of self-esteem. As described in detail previously [81], these findings highlight the importance of attending to psychological strengths in combination, instead of relying on a single predictor.

Our study has several strengths. The findings reported here provide evidence for increased meaning in life as a protective factor in internet addiction. Our findings also provide initial support for two specific psychological strengths working in tandem. The four research variables have been linked to many of the other personality constructs in the extant literature. As impulsivity might not largely decreased by psychological treatment, meaning and life, and self-esteem of psychological variables may instead be more effective in reducing addictive behaviors. This study contributes to diverse literatures, expanding the study of cognition, personality, clinical psychology, and psychiatry.

This study also has limitations. Due to the fact that the design is cross-sectional and correlational, it is limited in its usefulness in determining causality. In addition, our self-reported data may not be reliable, as it is subject to response bias. Because our examination did not control for relevant background predictors, we cannot be certain how well our results generalize to other groups. Future research investigating the same questions using an experimental design and truly random samples would address the limitations just discussed. Last, our results pertain only to meaning in life and self-esteem as protective factors against a general construction of internet addiction. Future research exploring additional personality traits as protective factors would yield more comprehensive insights into countering any specific dependent variables of behavior or substance addictions.

## Conclusions

In conclusion, the results of this study support a moderated mediation model of how the discussed constructs influence internet addiction. Empirical examination revealed a stronger indirect influence of impulsivity on internet addiction. Meaning in life and self-esteem can be a useful buffer against internet addiction for at-risk individuals who are highly impulsive. This research supports the utility of inspecting the individual difference mechanisms that might drive the relationship between impulsivity and internet addiction.

## Supporting Information

S1 File Supporting Information. (DOC)
