# Supplementary material for: The Relationship between Impulsivity and Internet Addiction in Chinese College Students: A Moderated Mediation Analysis of Meaning in Life and Self-Esteem
Source: PLoS One. 2015 Jul 14;10(7):e0131597. doi: 10.1371/journal.pone.0131597 (PMC4501566; doi:10.1371/journal.pone.0131597)
Supplement: S1 File — (DOC) [file pone.0131597.s001.doc]

Supporting Information

**S1 Dataset. Raw data of 1068 college students, which were analyzed in this study.**

**S1 Fig. The conceptual framework**


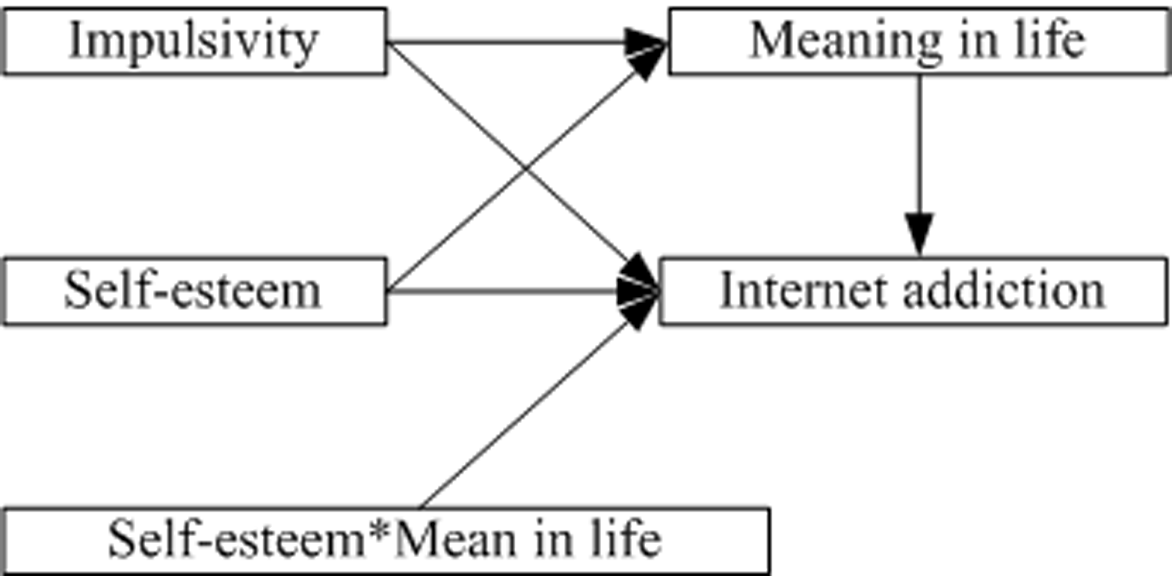


**S2 Fig. Standardized regression coefficients for paths within the mediation model. Explained variance: β, standardized regression coefficient**

**
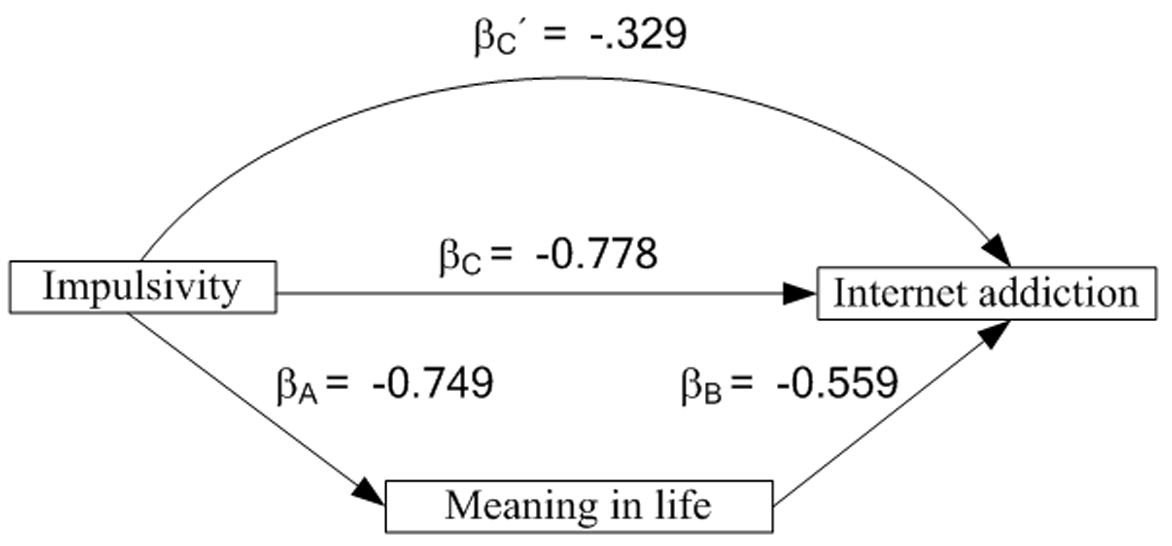
**

**S3 Fig. Regression lines for relations between meaning in life and Internet addict as moderated by self-esteem. (a 2-way interaction) . *b* = unstandardized regression coefficient (i.e., simple slope); *SD* = standard deviation**

**
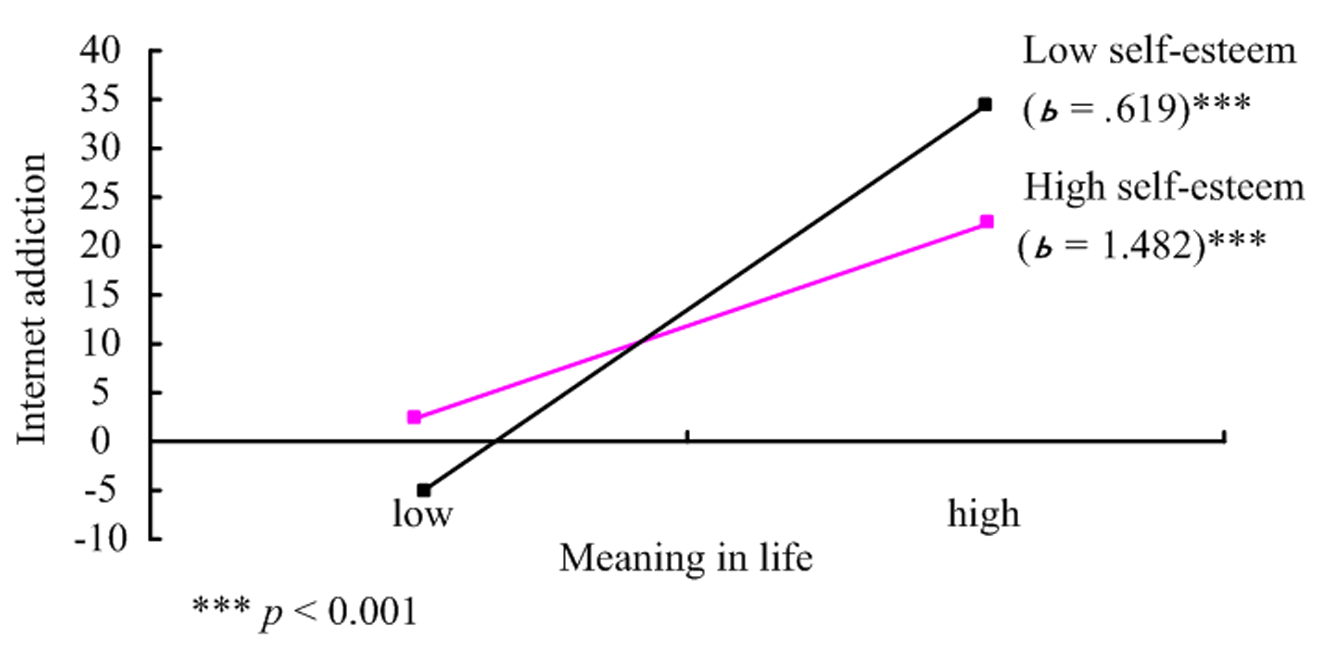
**

**S1 Table. Means, standard deviations, intercorrelations, and internal consistency between study variables**

|  | **1** | **2** | **3** | **4** |
| --- | --- | --- | --- | --- |
| **1 Internet addiction** | — |  |  |  |
| **2 Impulsivity** | -.781^**^ | — |  |  |
| **3 Meaning in life** | .846^**^ | -.751^**^ | — |  |
| **4 Self esteem** | .723^**^ | -.546^**^ | .647^**^ | — |
| ***M*** | 14.58 | 71.19 | 94.84 | 28.90 |
| ***SD*** | 1.742 | 9.802 | 13.617 | 4.131 |
| ***α* coefficient** | 0.730 | 0.787 | 0.795 | 0.789 |

Note: N = 1068; ^**^ *p* < 0.01

**S2 Table. Summary of hierarchical regression analysis for meaning in life in mediating the relationship between impulsivity and internet addiction**

**Explained variance: step 1, R^2^= .606; step 2, R^2^= .561; step 3, R^2^= .763; *B*, unstandardized regression coefficient; *SE*, standard error of *B*.**

|  | ***B*** | ***SE*** | ***t*** |
| --- | --- | --- | --- |
| ***PATH c (Step 1)*Internet addiction** | -0.139 | -.003 | -40.349^***^ |
| ***PATH a (Step 2)*meaning in life** | -1.403 | -.028 | -37.081^***^ |
| ***PATH c’+ b (Step 3)*Internet addiction** | Y=.076w  -.059x | .003.004 | 26.527^***^  -14.577^***^ |

Note: ^***^ *p* < 0.001

Explained variance: step 1, *R^2^* = .606; step 2, *R^2^* = .561; step 3, *R^2^* = .763; *B*, unstandardized regression coefficient; *SE*, standard error of *B*.

**S3 Table. The buffering effect of self-esteem on the relationship between meaning in life and internet addiction**

| **Step** |  | **1** | |  | **2** | |  | **3** | |  | **4** | |
| --- | --- | --- | --- | --- | --- | --- | --- | --- | --- | --- | --- | --- |
| **Predictor** |  | **Internet addiction** | |  | **meaning in life** | |  | **Internet addiction** | |  | **Internet addiction** | |
|  |  | ***b*** | ***t*** |  | ***b*** | ***t*** |  | ***b*** | ***t*** |  | ***b*** | ***t*** |
| **Impulsivity** |  | -.10 | -29.39^***^ |  | -.78 | -26.06^***^ |  | -0.05 | -14.33^***^ |  | -.04 | -13.87^***^ |
| **Self-esteem** |  | .178 | 22.48^***^ |  | 1.12 | 15.69^***^ |  | 0.11 | 15.03^***^ |  | .09 | 14.26^***^ |
| **meaning in life** |  |  |  |  |  |  |  | 0.81 | 20.42^***^ |  | .78 | 23.16^***^ |
| **meaning in life × Self-esteem** |  |  |  |  |  |  |  |  |  |  | -.09 | -19.83^***^ |
| ***△R^2^*** |  |  | |  |  | |  |  | |  | 0.05^*^ | |

Note: ^***^ *p* < 0.001, ^*^ *p* ≤ 0.05
